# Streptococcal infections, autoimmunity, and innate immune system in adult ADHD: A preliminary study

**DOI:** 10.1192/j.eurpsy.2022.853

**Published:** 2022-09-01

**Authors:** G. Di Girolamo, S. Peracchia, M. Boero, I.F. Bracco, F. Oliva

**Affiliations:** 1 University of Turin, Departiment Fo Neuroscience “rita Levi Montalcini”, Orbassano(TO), Italy; 2 University of Turin, Department Of Clinical And Biological Sciences, Orbassano (TO), Italy

## Abstract

**Introduction:**

High rate of streptococcus-like infections and related titers has been found in adult ADHD patients. No studies have expressively investigated innate immune system in ADHD patients.

**Objectives:**

To evaluate the relationship between streptococcal infections, autoimmunity and innate immune system in adult ADHD patients.

**Methods:**

The study sample consisted of adult DSM-5 ADHD outpatients referring to the adult ADHD center of “San Luigi Gonzaga” University Hospital and non-clinical adult controls recruited among general population (screened using Adult ADHD Self-Report Scale - ASRS-v.1). All titers were determined in patients’ plasma by specific microwell ELISA kits, whereas genetic polymorphisms were determined by PCR methodology. We compared anti-streptolysin O (ASO), anti-deoxyribonuclease B (anti-DNase B), and anti-basal ganglia antibodies (ABGA) titers of patients with those of controls. Data about history of previous streptococcus/ streptococcus-like infections were collected by ad-hoc form. Furthermore, to investigate the susceptibility to Gram+-borne infections of adult ADHD patients, due to innate immune system impairment, we also evaluated the polymorphism of Toll-like receptors 2, 4, and 9.

**Results:**

Although ADHD patients did not show higher rate of both previous infections (52.7% vs. 66.7%, p=.678) and ASO titers (18.2% vs. 0.0%, p=.577), they had really higher levels of anti-DNase B (85.5% vs. 16.7%, p=.001) and ABGA titers (78.2% vs. 33.3%, p=.036). Genetic analysis did not underline differences in polymorphism compared to general population (GENOME browser).

**Conclusions:**

The high association between previous streptococcal infections, basal ganglia autoimmunity among ADHD patients was confirmed. TLR polymorphism does not seem to be involved in this type of vulnerability.

**Disclosure:**

No significant relationships.

